# Contamination Transport in the Coastal Unconfined Aquifer under the Influences of Seawater Intrusion and Inland Freshwater Recharge—Laboratory Experiments and Numerical Simulations

**DOI:** 10.3390/ijerph18020762

**Published:** 2021-01-18

**Authors:** Qiaona Guo, Yue Zhao, Zili Hu, Mengjun Li

**Affiliations:** School of Earth Sciences and Engineering, Hohai University, Nanjing 211100, China; WHY961225@163.com (Y.Z.); CoweixongYytiupteud@gmail.com (Z.H.); 191609020028@hhu.edu.cn (M.L.)

**Keywords:** numerical simulation, laboratory experiment, contaminant transport, coastal heterogeneous unconfined aquifer

## Abstract

The coupled effect of seawater intrusion and inland freshwater recharge plays an important role in contamination transport in coastal heterogeneous aquifer. In this study, the effects of seawater intrusion and inland recharge on contamination transport were investigated by conducting laboratory experiments and numerical simulations. The laboratory tests were conducted in a sand tank considering two scenarios, namely the conditions of landward and seaward hydraulic gradients. The SEAWAT software was applied for validating the contaminant transport in coastal heterogeneous aquifer. The results indicated that the simulated seawater wedge and contours of the saltwater contaminant matched the observed ones well. The length of the seawater wedge in the scenario of seaward hydraulic gradient was smaller than that in the scenario of landward hydraulic gradient, which reflected that the large quantity of inland recharge have a negative effect on the invasion process of seawater. The plume moved mainly downward in the heterogeneous unconfined aquifer for both scenarios. The pollution plume became concave at the interface between each two layers, which was because the velocity of contaminant plume migration increased gradually from the upper layer to lower layer. The migration direction of the front of the plume was consistent with the direction of hydraulic gradient, which indicated that it was influenced by the water flowing. The maximum area of plume in the scenario of seaward hydraulic gradient was slightly smaller than that in the scenario of landward hydraulic gradient. The maximum area and vertical depth of the pollutant plume were sensitive to the hydraulic conductivity, dispersivity and contamination concentration. This study was of great significance to the controlling of pollution and utilization of freshwater resources in coastal areas.

## 1. Introduction

The coastal and estuarine areas are the most popular places for human, and most of the famous cities are located there in the world [1,2,3,4,5]. With the development of economy of human society, a myriad of coastal hydrogeological, engineering, biochemical, ecological and environmental problems arise. The problems include, for instance, seawater invasion, land subsidence, engineering structures stability and marine environment deterioration [5,6,7,8,9,10,11,12]. Among them, the environmental pollution caused by aquaculture is more serious, which is related to the formation of red tide [13]. Over the past 40 years, the aquaculture in the world increased by 8.7% every year, which was the fastest-growing food-producing sector [14]. Especially in recent years, the aquaculture has developed rapidly in coastal area, and the area of aquaculture is expanding. The seawater at the interface between saltwater and freshwater is extracted in large quantities for aquaculture; however, there is leakage in the process of aquaculture wastewater discharge, which leads to the pollution in coastal aquifer by seawater. Thus, understanding contamination transport caused by aquaculture in the aquifer is of great importance for managing and utilizing the freshwater resources in coastal zones.

Although the groundwater dynamics and hydrochemistry in coastal aquifers have been studied by scholars, there are relatively few reports on the contamination transport under the influence of hydrodynamic force. Most of the previous studies have focused on the time and space distribution of salinity in coastal zones, because they were often invaded by seawater [3,5,15,16,17,18,19,20]. There were two types of flow models simulating the mixing zone of seawater intrusion, consisting of the sharp interface model and mixing interface model [21,22,23,24,25,26,27]. Many analytical solutions were predominantly derived to describe the distribution of seawater intrusion in coastal aquifers (e.g., [23,28,29,30,31]). However, the analytical solutions were based on several hypothetical conditions. The numerical simulation were widely used to simulate seawater intrusion based on the mixing interface model using the variable-density flow and transport equations [32,33,34]. Notwithstanding, there were few studies considering the effect of seawater intrusion on pollutant transport. In addition, the tidal fluctuation and the groundwater dynamics in the coastal aquifer were very complex, which affected the spatial distribution and discharge rate of contamination.

In recent years, the laboratory experiment, analytical solution and numerical simulation were used to solve the problem of contamination migration in coastal zones [8,35,36,37,38,39,40,41,42,43,44,45,46]. The experimental method has been applied earlier to resolve the problem of pollutant migration in coastal aquifer. For example, Zhang et al. [35] and Zhang et al. [47] investigated the transport processes of contaminant under different conditions by laboratory experiments. Boufadel et al. [48] also conducted laboratory tests to study the nutrient transport in the beach aquifer, combining with the simulation method. In addition, an analytical solution was developed to explain directly the physical process in the ideal situation, in order to verify the model. Koohbor et al. [8] derived semi-analytical solutions describing the pollutant transport in the confined coastal aquifer under the condition of variable velocity field recently. Nevertheless, because of the complexity of boundary conditions, analytical approach can only be used to solve the contaminant transport under simple conditions. In reality, numerical solutions were more widely used to solve the problem of contaminant transport. For example, Guo et al. [41] developed the numerical model to simulate the contaminant transport at a gravel beach, combining with the field experiment. Shen et al. [46] considered contaminant transport affected by unstable flow in the beach aquifers by numerical simulation.

The contaminant transport in the beach was affected by many factors, including the tide fluctuation, variable density flow, pollutant density and aquifer heterogeneity [46,49,50,51,52]. The tracers were used to represent the conservative pollutants by the scholars. It was found that the contaminant was discharged into the sea through the subsurface of the beach with the groundwater flow, and the contour and migration path of the contaminant plume were influenced by the sea tide [41]. The contaminant transport was not only affected by the salt wedge in the low tide of intertidal zone, but also by the upper plume of saltwater within shallow zone. However, the density effect of contaminant was usually ignored. Bakhtyar et al. [52] further considered the solute plume transport affected by variable-density in beach aquifers. It was found that the discharge path and spread of plume were influenced by density difference. Besides, Geng and Boufadel [53] simulated the density-dependent subsurface flow and contaminant transport in shallow beach aquifers in response to wave and tide. The results reported that the plume migrated deeper and more seaward in the beach by the waves, compared to that under the condition of tide-only forcing.

However, in previous studies, there was a poor understanding of pollution under heterogeneous conditions in the coastal aquifer. The law of lithium tracer migration was investigated in a coastal gravel aquifer with two-layered structure [41]. Jang et al. [54] identified the factor of soil heterogeneity affecting nitrate reduction processes. Generally speaking, the previous studies have shown that the aquifer heterogeneity influenced the contaminant movement in a coastal aquifer. In addition, the spreading of contaminant transport in a coastal aquifer was affected by the inland water level oscillations, which was usually neglected. Liu et al. [55] considered the water level oscillation effect on the terrestrial pollutant transport at the inland boundary of the coastal aquifer. However, the studies of the influences of seawater intrusion and inland recharge on the presence of contamination in heterogeneous aquifer were scarce.

In the coastal areas, there may be problems such as saltwater leakage in the fish pond, seawater leakage through the pipeline, water quality deterioration, which lead to an obvious contaminated area occurring in the coastal area. Up to now, there is no knowledge of how the saltwater pollution affects groundwater in heterogeneous coastal zone. The influence of inland freshwater recharge on pollution distribution was seldom considered. In this work, the goal of the study was to investigate the contaminant transport in heterogeneous coastal aquifer with laboratory experiment and numerical simulation. The combined effects of seawater intrusion, inland freshwater recharge and aquifer heterogeneity on the contaminant transport were considered. The landward and seaward hydraulic gradients were studied. The contaminant source was saltwater placed at the phreatic surface of the aquifer in both scenarios. The law of contaminant transport in the heterogeneous aquifer was considered, and the experimental and numerical results were compared to study the contaminant transport for different conditions of inland recharge.

## 2. Material and Methods

### 2.1. Laboratory Experiment

**Figure 1:**
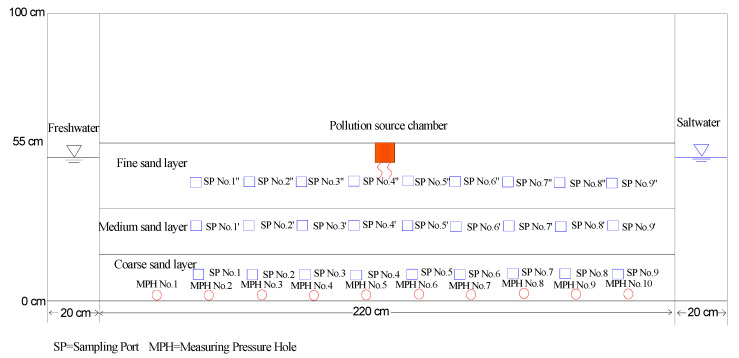
Schematic diagram of the test model.

The laboratory tank has a length of 260 cm, width of 30 cm and height of 100 cm, which was made by plexiglass (Figure 1). The flow tank was composed of four parts, which were seawater chamber, seepage chamber, freshwater chamber and pollution source chamber. The freshwater chamber and seawater chamber on both sides were separated from the seepage chamber in the middle zone by plates made of polyvinyl chloride material. The PVC board was evenly drilled with holes of the same size. The geotextile was pasted on the surface of the PVC plate to avoid the sand flowing from the seepage chamber to seawater chamber or freshwater chamber. The water reservoirs connected with the bottom of the seawater chamber and freshwater chamber, which were made by the reinforced organic plastic plate. The seawater and freshwater were pumped into the seawater chamber and freshwater chamber by the peristaltic pumps, respectively. The freshwater chamber on the left side and the seawater chamber on the right side represented the inland boundary and sea boundary, respectively. The pollution source chamber was located at the place of 106 cm far away from the freshwater chamber, which was made of transparent organic plastic plate. The dimension of the plate used was 30 cm (length) × 5 cm (width) × 20 cm (height). The bottom of it was evenly distributed with circular holes of the same size. A layer of geotextile was pasted on the bottom of it to prevent the sand flowing into the pollution source chamber. The bottom of the pollution source chamber was located at the groundwater level in the sand aquifer of the tank. The pollutant with constant concentration was pumped into the pollution source chamber by the peristaltic pump at a constant speed. The seepage chamber was filled with fine sand, medium sand and coarse sand from upper layer to lower layer. The thicknesses of the three layers were 23 cm, 16 cm and 16 cm, respectively. Each layer was filled with water and compacted as much as possible to remove the air. The constant head permeability tests were conducted to estimate the hydraulic conductivity (*K*). The values of *K* were 2 m/d, 24.95 m/d and 59.5 m/d for the fine sand, medium sand and coarse sand, respectively.

There were ten pressure taps arranging at the bottom of the sand tank, which were used to measure the groundwater level variation in the aquifer. The distance between each two pressure taps was 20 cm. The pressure tap was connected with a transparent PU pipe, which was connected with an acrylic transparent glass pipe on the pressure plate. A 0.2 mm filter screen was arranged at the pressure tap to prevent the sand particles from flowing into the PU pipe and blocking the pipe. A ruler was installed on the pressure plate to record the groundwater head in the sand tank at different times during the experiment. The sampling ports were placed on the back of the sand tank, and there were nine sampling ports in each layer. The interval between each two sampling ports was 20 cm. More details about the experiment device can be found in Guo et al. [56].

During the test, the water samples were taken out from the sampling ports, and the concentration of Cl^−^ in the samples was determined by DDS-307 conductivity meter (Hongyi Instrument Co., Ltd, Shanghai, China). Two sets of experiments were considered for different direction of hydraulic gradient slopes. In the first experiment (Scenario 1), the water head at the inland boundary and seawater level at the sea boundary were fixed at 46 cm and 47 cm, respectively. In the second experiment (Scenario 2), the inland head and seawater level were set to be 48 cm and 47 cm, respectively. During the process of experiment, the water level at each measuring pressure hole and the salt concentration at each sampling port were detected. The freshwater was made by deionized water in the laboratory, and the density of it was 1.0 × 10^3^ kg/m^3^. The concentration of saltwater and density were 35.0 g/L and 1.02 × 10^3^ kg/m^3^, respectively. The saltwater in the right chamber was dissolved using bright blue, with the concentration of 2.0 g/L, in order to trace the seawater intrusion process during the experiment. The contamination injected in the pollution source chamber was saltwater. The concentration of salinity was 35.0 g/L. The tracer of carmine was added in the saltwater to trace the movement of contamination. The concentration of it was 1.0 g/L.

Before starting the contamination transport experiments, the sand flume was filled with the freshwater. Then, the seawater flowed from the water reservoir to the seawater chamber, and the overflow flowed through the outlet of the chamber. Subsequently, the stable water flow was formed and the saltwater gradually entered the tank from the seawater chamber. The equilibrium status between the saltwater and freshwater was considered to be reached, when the length of the seawater wedge did not change within 5 min. The peristaltic pump was activated to inject the contamination into the chamber. The flow rate was set to 0.44 m/h by controlling the peristaltic pump. At this time, the contaminant moved under the hydraulic gradient and density difference between saltwater and freshwater. Finally, the experiment finished as the contaminant intersected with the interface between the saltwater and freshwater when it migrated in the coarse sand layer. The experiment process for the Scenario 1 was similar to that of Scenario 2. During the experiments, the Canon digital camera (IXUS 175) (Canon corporation, Tokyo, Japan). was used to record the process of saltwater wedge and pollutant migration. It has the pixel of 3648 × 2736 and the function of 8 × optical zoom. The interval time for measuring the concentration of salt was 5 min. After the experiment, the contaminant plume and saltwater wedge in the aquifer were determined by a MATLAB code.

### 2.2. Numerical Model and Procedure

The code SEAWAT version 4 was used to establish the numerical simulation model of solute transport in groundwater flow, which was based on the simulation model MODFLOW of finite difference, considering the effect of density on groundwater flow [57,58]. The software was widely used for modelling the process of saltwater intrusion and submarine groundwater discharge (e.g., [5,50,59,60,61,62]). The details on the coupling governing equations for the variable density groundwater flow and solute transport were shown in Guo et al. [5].

The area in the numerical model was a heterogeneous layered unconfined aquifer. The parameters obtained from the laboratory experiments were followed as close as possible during the setting of the numerical simulation. A two-dimensional, vertical cross section was built. The length and height of the section were 220 cm and 55 cm, respectively. The domain of model was divided as 24 layers and 5000 columns. A free surface boundary was defined on the upper part. It was assumed that there was no flow on the bottom boundary condition of the numerical model. The inland boundary head and seawater level were set to 46 cm and 47 cm on the left and right boundaries in Scenario 1. The left inland head and right seawater level were fixed at 48 cm and 47 cm in Scenario 2. The concentrations of seawater and freshwater were equal to 35 g/L and 0 g/L, respectively. Supposing that the contaminant on the water table of the model was uniformly distributed at the distance between 1.04 m and 1.08 m. The constant concentration of contamination was set to 35 g/L in Scenario 1 and Scenario 2. During the simulation, the constant contamination infiltration rate along the bottom of the chamber was 0.44 m/h. The simulated time was 100 min and 110 min, respectively, for Scenario 1 and Scenario 2. During the simulation, the time step was 60 s for both Scenarios.

The parameters in the heterogeneous aquifer were estimated, through calibrating the observed head and salinity using the trial-and-error method repeatedly. The calibration process was to adjust the parameters values until the difference between the simulated and observed values was minimized at the sampling ports. The horizontal hydraulic conductivity was supposed to be same to the vertical hydraulic conductivity in each layer of the aquifer. The estimated hydraulic conductivities were 4.0 m/d, 25.0 m/d and 60.0 m/d from the top layer to lower layer. By fitting the water level and salinity, the parameter values of the aquifer were obtained and listed in Table 1.

## 3. Results and Discussions

### 3.1. Salinity of Basic Scenarios

**Figure 2:**
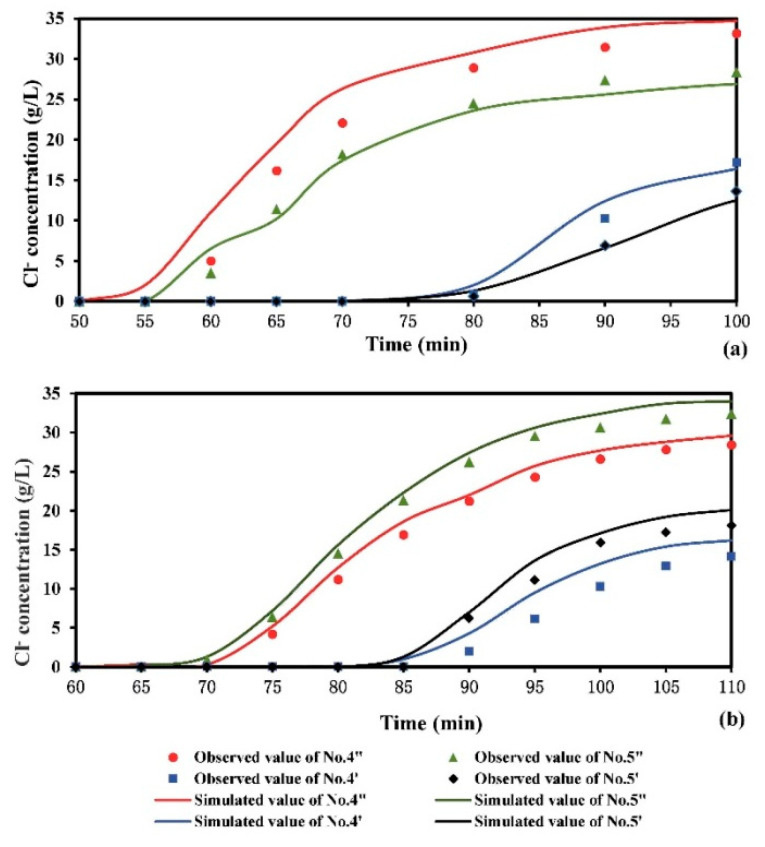
Comparison of simulated and observed Cl^−^ concentrations over time at selected observation points (No. 4′, No. 5′, No. 4′′ and No. 5′′) for (**a**) scenario 1 and (**b**) scenario 2.

Figure 2a reported the experimental Cl^−^ concentrations versus the simulated ones at the four observation points for Scenario 1. It can be seen that the simulated Cl^−^ concentrations have a good correlation with the observed ones. The correlation coefficient (R^2^) ranged from 0.982 to 0.998. The calculated confidence interval was 95%. The observed and simulated Cl^−^ concentrations values at the points increased with time dramatically initially, and then they increased slowly and became stable. The Cl^−^ concentrations at points No. 4′′ and No. 5′′ in the fine sand layer were higher than those at points No. 4′ and No. 5′ in the medium sand layer. It indicated that the contaminant transport in the medium sand layer lagged behind that in the fine sand layer. The migration time of contaminant was about 23 min from the upper layer to middle layer. In steady state, the Cl^−^ concentration at left point No. 4′′ was 34.8 g/L, which was higher than that at right point No. 5′′ in the upper layer. In the middle layer, the Cl^−^ concentration at left point No. 4′ was larger than that at right point No. 5′, which was similar to the fine sand layer. It revealed that the contaminant concentration distribution was affected by the flowing from the sea to inland.

The comparison between the experimental Cl^−^ concentrations and numerical values at the selected observation points for scenario 2 was shown in Figure 2b. Generally, the simulated Cl^−^ concentrations and observed ones agreed well. The calculated confidence interval was 95%. The average significant correlation coefficient (R^2^) were between 0.983 and 0.997. The simulated Cl^−^ concentration at point No. 5′′ was larger than the observed one. The reason might be that there was a small amount of freshwater in the sample point during the experiment, which diluted the concentration of sample. Similar to scenario 1, the Cl^−^ concentrations in the upper layer were higher than those in the middle layer for scenario 2. The contaminant transport time was about 20 min from the upper layer to middle layer. The Cl^−^ concentration at left point No. 4′′ was lower than that at right point No. 5′′ for the fine sand layer in steady state. Similar to the fine sand layer, the Cl^−^ concentration at left point No. 4′ was lower than that at right point No. 5′ in the medium sand layer. Compared with Figure 2a, one can see that the maximum value of Cl^−^ concentration was 32.7 g/L, which was lower than that of the scenario 1. It indicated that the contaminant concentration was diluted by the water flow, because of the hydraulic gradient slope from the inland to sea.

### 3.2. Transport of Contamination in Heterogeneous Aquifer

**Figure 3:**
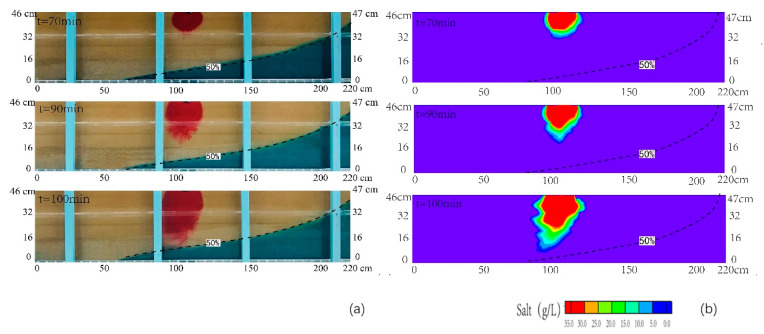
Observed (**a**) and simulated (**b**) contamination concentration distributions in the cross section for scenario 1. The black dashed line denoted the contour line with the concentration of 0.5 time of seawater concentration.

Figure 3a,b presented the comparison between the transient observed and simulated contours of the contaminant of salinity at various times after the injection (the time of 50 min) for scenario 1. The simulated results have a good correlation with the observed data from laboratory tests. It indicated that the simulated results were reliable. When the invasion of seawater reaches the equilibrium status, the horizontal length of the 50% isoline of the observed seawater wedge can reach 145 cm. Figure 3 reported that the plume mainly moved downward at different time. The freshwater and contaminant moved along the interface of the seawater wedge, which were influenced by the vertical velocity coupled with horizontal velocity from sea to inland. The salinity concentration reached the highest value around the infiltration area. The transition area at the edge of pollution plume was very narrow. During the time between 50 and 70 min, the plume moved downward and laterally, and the semi-elliptical shape was formed in the upper layer. Due to the higher velocity formed directly below the interface between upper layer and middle layer, the pollution plume became concave at the interface. Then, the plume moved downward in the medium sand layer during the time ranging from 70 to 90 min. At the time of 90 min, the plume began to migrate into the coarse sand layer. The pollution plume became concave at the interface of middle layer and bottom layer. At the time of 100 min, the simulated result showed that the plume migrated downward and landward with the water flowing in the coarse sand layer, which was consistent with the observed one in the laboratory. When the salinity pollution plume approached the saltwater interface, the front of it was more diffuse. It was in line with the study of Zhang et al. (2002). Finally, the observed seawater-contamination plume intersected with the seawater wedge at the time of 100 min.

**Figure 4:**
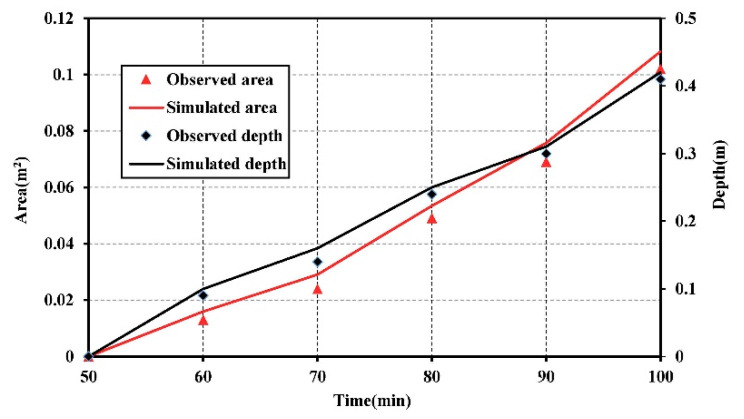
The observed and simulated seawater contamination plume area and maximum vertical pollution depth over time for scenario 1.

In this study, the saltwater plume concentration of 3.5 g/L was used as the threshold. The pollution plume area was defined by the area surrounded by the contour with concentration of 3.5 g/L and the upper boundary. Figure 4 demonstrated the observed and simulated seawater contamination plume area and maximum vertical pollution depth over time for scenario 1. The simulated plume area and maximum vertical pollution depth were, in general, higher than the observed ones. This might be due to the lighter tracer diluted by the freshwater during the experiment. The area of plume was zero at the initial time of 50 min. As the time was increased to 70 min, the seawater-contaminated area expanded linearly and the maximum vertical pollution depth reached 0.16 m. When the plume moved into the medium sand layer, the plume migration ability increased as the velocity increased. It caused the saltwater contaminated area expanding. The variation rate of area in the medium sand layer was two times of that in the fine sand layer. The maximum vertical pollution depth was 0.3 m at the time of 90 min. Then the plume migrated into the coarse sand layer. Because of the influence of the high velocity in the coarse sand, the area of pollution plume and the maximum vertical pollution depth increased. The variation rate of area in the coarse sand layer can reach 1.5 times of that in the medium sand layer. At the time of 100 min, the area of plume and maximum vertical pollution depth substantially increased to 0.1 m^2^ and 0.41 m. The layered aquifer was seriously polluted by the saltwater pollutants in this scenario.

**Figure 5:**
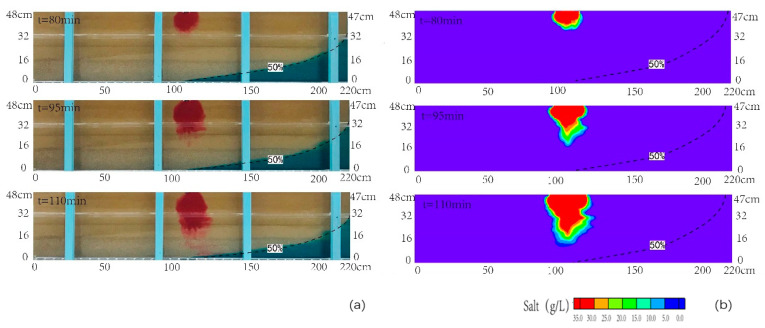
Observed (**a**) and simulated (**b**) contamination concentration distributions of the cross section for scenario 2. The black dashed line denoted the contour line with the concentration of 0.5 time of seawater concentration.

Figure 5a,b illustrated the observed and simulated plume movement at various times for scenario 2. The results showed that the simulated motion of seawater-contamination plume matched the observed one well. The observed length of the 50% isoline of the seawater wedge was 110 cm, which was smaller than that of scenario 1. It was because that the head at inland boundary was higher than the seawater level, and hydraulic gradient slope was inclined to the sea. It reflected that the large quantity of inland recharge had a negative impact on the seawater intrusion process in the layered aquifer, which was of great significance to the prevention and controlling of seawater intrusion in coastal areas [5]. From Figure 5, one can see that the plume migrated in the upper layer and the shape of it was semi-elliptic during the period of 60 and 80 min. The concentration of the plume was highest in the infiltration area, and it decreased gradually on both sides. Similar to scenario 1, the plume became concave at the interface of the upper layer and middle layer. Then, the plume moved downward in the medium sand layer at the time between 80 and 95 min. There was a narrow transition region at the edge of the pollution plume, which indicated that the concentration of plume decreased gradually in the process of migration. It was consistent with the observation by Sun et al., (2019). Subsequently, the plume moved into the bottom layer. The front of the simulated plume migrated with flow towards the sea boundary at the time of 110 min, which was consistent with that of the laboratory experiment. There was no preferential flow in the numerical simulation due to the homogeneity of the sand layers, which was different from that of the experiment.

**Figure 6:**
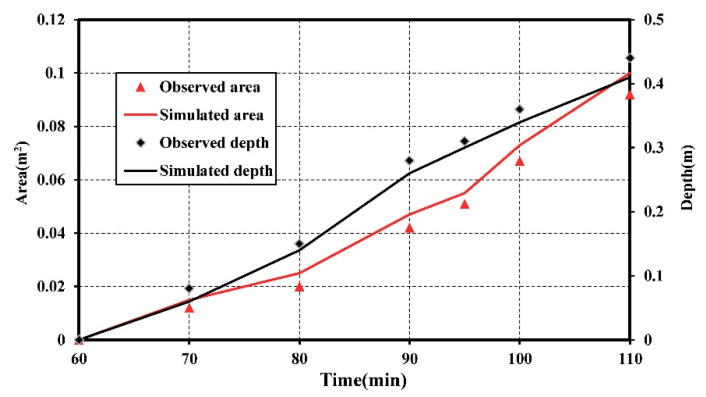
The observed and simulated seawater contamination plume area and maximum vertical pollution depth over time for scenario 2.

Figure 6 presented the observed and simulated seawater contamination plume area and maximum vertical pollution depth over time for scenario 2. The simulated plume area and maximum vertical pollution depth were slightly larger than the observed values, which was similar to scenario 1. The area of plume and maximum vertical pollution depth increased slowly with time in the upper layer. The area of plume increased by 0.02 m^2^ for a 0.16 m increase in the maximum vertical pollution depth. Then, the area of plume and the maximum vertical pollution depth began to increase rapidly with respect to time, due to the higher velocity in the medium sand layer. The area of plume increased by 0.03 m^2^ for a 0.16 m increase in the depth, which indicated that the variation rate of area increased in the medium sand layer compared to that of the fine sand layer. When the plume migrated into the coarse sand layer, the area of plume and the maximum vertical pollution depth increased more rapidly with respect to time in the medium sand layer. The variation rate of area in the coarse sand layer reached 1.4 times of that in the medium sand layer. At the time of 110 min, the area of plume increased to 0.09 m^2^, which was slightly smaller than that of scenario 1. It demonstrated that the inland recharge diluted the concentration of contamination plume.

### 3.3. Sensitivity Analysis

The hydraulic conductivity, dispersivity and concentration of contamination were important parameters for solute transport in the heterogeneous aquifer [56]. Therefore, the sensitivities of saltwater contamination transported in the aquifer to the model parameters were analyzed in the scenario of landward hydraulic gradient. The area and velocity of the plume diffusion were discussed by changing the above parameters. It should be noted that the parameter values for analyzing the sensitivity were increased or decreased, whereas the values of the other parameters were not changed, which was shown in Table 1.

**Figure 7:**
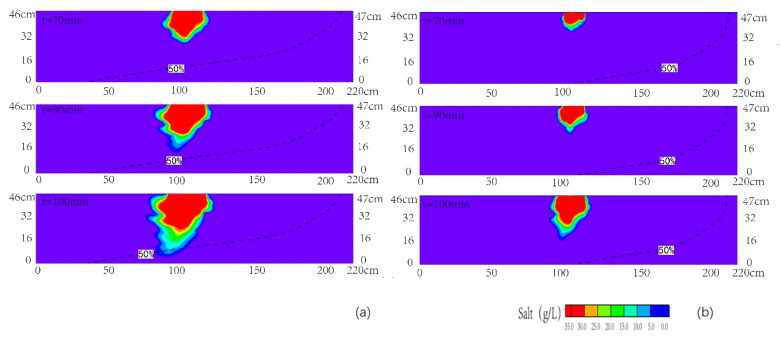
Simulated spatial saltwater contamination concentration distributions for (**a**) increasing hydraulic conductivities and (**b**) decreasing hydraulic conductivities at different times. The black dashed line denoted the contour line with the concentration of 0.5 time of seawater concentration.

Firstly, the influence of the hydraulic conductivity K on plume movement was investigated. Figure 7a displayed the simulated spatial saltwater concentration distributions with time by the new model, as the values of *K* were raised to 8.0 m/d, 50.0 m/d and 120.0 m/d for the upper layer, middle layer and bottom layer, respectively. Compared with Figure 3b, Figure 7a showed that the match to the observed contours of the saltwater contamination in the new simulation was worse, compared with the simulated ones in the scenario 1. It was found that more saltwater contamination than in reality migrated into the aquifer of the new model. The region of transition zone in the front edge of plume in the new simulation became larger, compared with that in the basic model, which was because the velocities were larger with higher hydraulic conductivities. The dispersion of the saltwater plume became stronger and the plume migrated more downward and landward. Increasing the hydraulic conductivity increased remarkably the area of the plume in the heterogeneous aquifer. The maximum area of pollution plume simulated by the new model reached 0.14 m^2^, which was larger than that of scenario 1.

As the values of K were reduced to 2.0 m/d, 12.5 m/d and 30.0 m/d for the upper layer, middle layer and bottom layer, respectively. Figure 7b showed that the match to the observed contours of the saltwater contamination was considerably worse, compared with the simulated ones in the Figure 3b of scenario 1. Obviously, the maximum vertical pollution depth decreased when the hydraulic conductivities of the heterogeneous aquifer decreased, which indicated that the hydraulic conductivities for sensitivity analysis were too low. Additionally, with the lower hydraulic conductivities in each layer, the velocity of plume migration became slowly. However, the migration direction of pollution plume remained downward and landward. The area of pollution plume simulated by the new model increased slowly, and the maximum value of it reached 0.06 m^2^, which was smaller than that of scenario 1. Therefore, one can conclude that increasing and decreasing the hydraulic conductivities by the new models were not able to reproduce the spatial distributions of saltwater contamination concentration.

**Figure 8:**
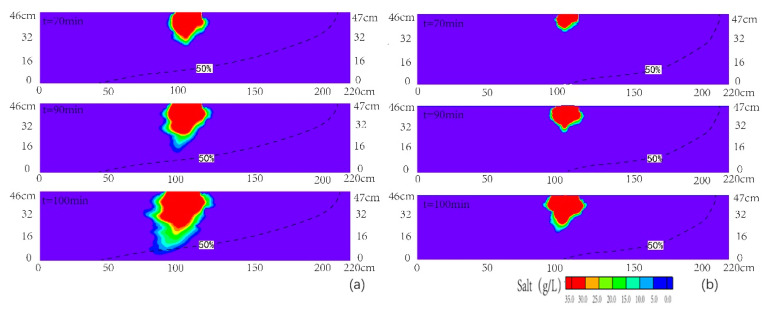
Simulated spatial saltwater contamination concentration distributions for (**a**) increasing dispersivity and (**b**) decreasing dispersivity at different times. The black dashed line denoted the contour line with the concentration of 0.5 time of seawater concentration.

Secondly, the sensitivity of the plume transport to the dispersivity was explored for two new models, because the dispersivity was important for solute transport. The variation of contours of the saltwater contamination was used for analyzing sensitivity with respect to the dispersivity. When the value of *α_L_* increased to 0.5 m and the ratio *α_T_*/*α_L_* was 0.1, Figure 8a showed that more saltwater contamination migrated into the aquifer of the new model than that of basic Scenario. The simulated maximum vertical pollution depth was larger, compared with that in the basic model, which could be because high dispersivity induced widening of the transition zone. The area of pollution plume simulated by the new model was 0.135 m^2^, which was larger than that of scenario 1. It indicated that higher dispersion has great influence on plume transport. However, there was no change in the direction of pollutant migration.

When the value of *α_L_* decreased to 0.05 m and the ratio *α_T_*/*α_L_* was 0.1, Figure 8b showed that the area simulated by the new model could not match the observed ones at different times, compared with Figure 3b. It depicted that the area of pollution plume simulated by the new model was 0.061 m^2^, which was much smaller than that of scenario 1. It indicated that decreasing the dispersion would lead to the slow migration velocity of plume. Overall, changes in dispersivity have effects on the plume transport. 

**Figure 9:**
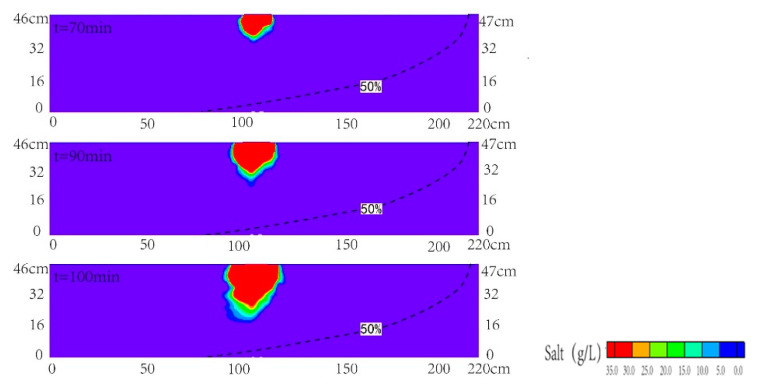
Simulated spatial saltwater contamination concentration distributions at different times when the injected saltwater concentration decreased to 15 g/L. The black dashed line denoted the contour line with the concentration of 0.5 time of seawater concentration.

Finally, the sensitivity of the plume transport to the concentration of contamination was analyzed. The saltwater concentration was reduced from 35 g/L to 15 g/L in the new model. Figure 9 showed that the fitting to the observed plume was worse, compared with that of basic scenario in Figure 3b. One can see that the area of pollutant plume and maximum vertical pollution depth decreased sharply, whereas the movement of the plume decreased slightly in the horizontal direction of the new model, compared with the simulated ones in scenario 1. It was because the dispersion was too small. At the time of 100 min, the simulated area of plume was 0.07 m^2^, which was smaller than that of basic Scenario. However, the direction of pollutant plume migration was downstream and landward in the aquifer. Therefore, the range of plume was sensitivity to the concentration of contamination.

## 4. Conclusions

The investigation of contaminant transport in heterogeneous unconfined aquifer of coastal zone is insufficient in previous studies. In this paper, both laboratory experiments and numerical simulations were applied to research the contamination transport in the heterogeneous unconfined aquifer with two Scenarios of hydraulic gradients. The following conclusions can be summarized as follows:

The simulated spatial distribution of seawater wedge and contaminant plume agreed with the observed ones. The comparison between the experimental Cl^−^ concentrations and numerical values at the selected observation points matched well. The plume moved mainly downward in the heterogeneous unconfined aquifer for both Scenarios, which became concave at the interface between each two layers. It was because the velocity of contaminant plume migration increased gradually from the upper layer to lower layer. When the salinity pollution plume approached the saltwater interface, the front of it was more diffuse. The migration direction of the front of the plume was consistent with the direction of hydraulic gradient, which indicated that it was influenced by the water flowing.

The maximum area of plume in the Scenario of seaward hydraulic gradient was 0.09 m^2^, which was slightly smaller than that in the Scenario of landward hydraulic gradient. It indicated that the larger inland recharge diluted the contamination plume. The maximum area and vertical depth of pollutant plume were sensitive to the hydraulic conductivity, dispersivity and contamination concentration. In this work, the contaminant plume movement in the heterogeneous aquifer with downward increasing of hydraulic conductivity was considered. In future, more cases should be studied, such as the aquifer where the hydraulic conductivity decreases with depth.

## Figures and Tables

**Table 1:** Model parameters and their values used in the numerical simulations.

Parameter	Definition	Unit	Value
*K*	Hydraulic conductivity	m/d	4.0 (fine sand layer)
			25.0 (medium sand layer)
			60.0 (coarse sand layer)
*μ*	Specific yield	-	0.2 (fine sand layer)
			0.25 (medium sand layer)
			0.30 (coarse sand layer)
*S_S_*	Specific storage	1/m	10^−5^
*ρ_f_*	Density of the freshwater	kg/m^3^	1.0 × 10^3^
*ρ*	Density of the seawater	kg/m^3^	1.02 × 10^3^
*n*	porosity	-	0.3
*α_T_*	Longitudinal dispersivity	m	0.1
	Transverse dispersivity	m	0.01
*τD_m_*	Molecular diffusion coefficient in porous media	m^2^/s	10^−9^
*t*	Time period	min	100 for scenario 1
			110 for scenario 2
